# Psychosocial stressors and cognitive function: An analysis using data from the English longitudinal study of ageing

**DOI:** 10.1016/j.tjpad.2025.100232

**Published:** 2025-06-17

**Authors:** Jiahao Li, Natalia Ortí-Casañ, Irem Bayraktaroglu, Giulia Mozzanica, Feng Zhang, Jocelien D.A. Olivier, Ulrich L.M. Eisel

**Affiliations:** a Department of Molecular Neurobiology, Groningen Institute of Evolutionary Life Science (GELIFES), University of Groningen, Groningen, The Netherlands; b Department of Neurology, the Second Affiliated Hospital of Zhejiang University School of Medicine, Hangzhou, China

**Keywords:** Psychosocial stressors, Cognitive decline, Executive function, Older adults, Longitudinal study, ELSA

## Abstract

**Background:**

Growing evidence suggests that psychosocial stressors—such as financial strain, caregiving responsibilities, disability, and limiting long-term illnesses—may contribute to accelerated cognitive decline in older adults. However, the heterogeneity of stressor profiles and their distinct impact on specific cognitive domains remain poorly understood.

**Objective:**

To examine the associations between varying burdens of psychosocial stressors and cognitive function over a 10-year period using data from the English Longitudinal Study of Ageing (ELSA).

**Methods:**

We used longitudinal data from wave 4 (2008–2009) to wave 9 (2018–2019) of ELSA, comprising 10,893 participants aged ≥50 years at baseline who had valid measurements of psychosocial stressors and cognitive outcomes. Psychosocial stressors—financial strain, caregiving, disability, and limiting long-term illness—were assessed as binary indicators and summed into three categories (No Stressors, One Stressor, Multiple Stressors). Cognitive function was assessed using an overall global cognition score and scores of three specific domains: memory, executive function, and orientation. Baseline associations were examined via multiple linear regression, while linear mixed-effects models evaluated longitudinal trajectories of cognitive change. All models were progressively adjusted for demographic, lifestyle, and clinical covariates.

**Results:**

At baseline, participants reporting multiple stressors (18.2 % of the sample) had significantly lower global cognitive and executive function scores compared to those with no stressors (43.3 %). Over the 10-year follow-up, a higher stress burden predicted faster declines in global cognition, memory, and executive function. These associations remained robust after adjusting for sociodemographic characteristics, health behaviors, and chronic conditions. Random intercept and random slope models yielded consistent findings, indicating a dose–response relationship between stress burden and cognitive deterioration.

**Conclusion:**

Older adults experiencing multiple psychosocial stressors face an elevated risk of both lower initial cognitive function and accelerated decline over time. These findings underscore the importance of identifying and mitigating modifiable stressors—such as financial strain and caregiving demands—to potentially preserve cognitive health in later life. Interventions tailored to individuals with higher stress burdens may be especially beneficial in slowing cognitive deterioration.

## 1 Introduction

Psychosocial stressors, encompassing factors such as financial strain, social isolation and caregiving responsibilities, have been shown to exert far-reaching implications on physical and mental health, including cognitive function [1,2]. Chronic psychosocial stress (hereafter referred to as “stress”) can lead to physiological and psychological dysregulation, which may accelerate neurocognitive decline [3,4]. Although various mechanisms have been proposed—for instance, altered hypothalamic–pituitary–adrenal axis activity [5], increased inflammatory markers [6], or heightened cardiovascular reactivity [7]—these findings provide only a partial explanation. Our understanding remains fragmented when it comes to identifying distinct stressor profiles in older adults and clarifying how these relate to cognitive health outcomes [2].

Stress has been frequently highlighted as a modifiable risk factor for diverse age-related conditions, ranging from depressive symptoms to cardiovascular and metabolic diseases [[8], [9], [10]]. Similarly, stress-related biological pathways—such as those involving cortisol or inflammatory mechanisms—have been implicated in cognitive decline [11,12]. However, the assumption that “stress indisputably disrupts cognition” may be overly simplistic, as it overlooks substantial interindividual variation in how psychosocial stressors are experienced and expressed among older adults [[13], [14], [15]]. This heterogeneity in stress exposure and biological response likely contributes to the inconsistent findings reported in the literature [[16], [17], [18], [19], [20]]. For instance, some studies suggest that chronic stress impairs memory, whereas others report no association or even short-term cognitive benefits under acute stress conditions. These mixed findings highlight the need to move beyond a one-dimensional view of stress and instead consider how different types of psychosocial stressors may interact and accumulate to affect cognitive outcomes.

In older populations, understanding these processes is imperative, given rising life expectancies and the health challenges associated with an ageing demographic [21,22]. Financial constraints, caregiving responsibilities, chronic illness, and disability are all prevalent in later life [[23], [24], [25], [26]]. Each of these experiences has the potential to compound long-standing life stressors, ultimately influencing cognitive trajectories through neurobiological mechanisms such as neuroinflammation or altered neuroendocrine function [2,4,[27], [28], [29], [30]]. While evidence suggests that accumulated life stress may be detrimental to cognitive function [2,4,30], systematic investigations into the distinct psychosocial stressor patterns that drive cognitive decline remain sparse, possibly partially due to the limited availability of comprehensive longitudinal data.

Additionally, genetic predispositions and individual-level factors contribute to the variability in stress responses and cognitive resilience [31,32]. This complexity implies that a one-size-fits-all framework may be insufficient to capture the nuance of psychosocial stressors, particularly among older adults. Rather, an approach that accounts for the coexistence of different stressor types—social, financial, and health-related—alongside biological and psychological moderators might yield a more precise understanding of which individuals or subgroups are most vulnerable to cognitive decline [33,34].

Methodological developments such as ordinal classification model (OCM) present new avenues for classifying individuals into subgroups based on multiple indicators [35,36], rather than examining each stressor in isolation. By evaluating how various psychosocial stressors cluster together in older adults, OCM can offer a nuanced perspective on how these distinct profiles relate to cognitive trajectories. This population-level approach can help reduce the impact of individual heterogeneity in the stress–cognition relationship and potentially guide targeted preventive and therapeutic interventions. Identifying subgroups at elevated risk for cognitive decline due to compounded psychosocial stressors could inform more personalized healthcare strategies that ultimately enhance clinical outcomes and resource allocation. In this context, our study aimed to classify and quantify distinct profiles of psychosocial stressors in a United Kingdom-based sample of older adults from the English Longitudinal Study of Ageing (ELSA), and to determine how these profiles are longitudinally associated with cognitive function.

## 2 Materials and Methods

### 2.1 Study design

This prospective cohort study utilized fully anonymized data from the ELSA, a nationally representative, multidisciplinary prospective observational study of individuals in England aged 50 years and older [37,38]. For the present analyses, we used data from wave 4 (2008-2009) as the baseline and followed participants through to wave 5-9 (2010-2019). Data collection involved in-home assessments through computer-assisted personal interviews (CAPI) and self-completion questionnaires every two years. All participants provided written informed consent, and ethical approval was obtained from the National Research Ethics Service (London Multicentre Research Ethics Committee) [37], in accordance with the Declaration of Helsinki. Detailed information on ELSA’s design and data collection procedures is available in Steptoe et al [37]. From wave 4, participants with missing or invalid data and those previously diagnosed with dementia or Alzheimer’s disease were excluded. The remaining individuals had data available on psychosocial stressors and cognitive outcome measures, forming the analytic sample.

### 2.2 Exposures

Building on prior research identifying relevant risks among older adults [[39], [40], [41]] and following the definition of psychosocial stressors proposed by Odessa et al [42], we assessed four psychosocial stressors occurring at wave 4. Each stressor was treated as a binary indicator (presence vs. absence), and an overall psychosocial stress score was calculated by summing these indicators, resulting in a possible range of 0 to 4. For the present analyses, we employed OCM to categorize participants into three groups based on their total stressor score: No Stressors (0), One Stressor (1), or Multiple Stressors (≥2). The four stressors were defined as follows:

1. Financial Strain: Binary indicator of perceived chance of not having enough financial resources in the future to meet needs; categorized by 0; 1–39; 40–60; 61–99; 100 % and dichotomized at > 60 %. The higher the percentage, the higher the belief of having insufficient resources and, thus, the higher the stress experience.
2. Caregiving: Binary indicator of being an informal caregiver within the past week to an adult who is sick or frail, or being a caregiver in receipt of Carer’s Allowance in the last month.
3. Disability: Binary indicator of having more than one mobility difficulty (e.g., walking 100 yards; sitting 2-hours; rising from chairs after sitting long periods; climbing stairs; stooping, kneeling, or crouching; reaching or extending arms above shoulders; pulling or pushing large objects; lifting or carrying objects over 10 lb; picking up a 5p coin).
4. Limiting long-term illness: Binary indicator of a long-standing illness or health condition that limits activities.

### 2.3 Outcomes

Cognitive function was assessed across waves 4 to 9 (2008–2019) and included measures of memory, executive function and orientation scores. Each participant's memory function was assessed using immediate and delayed recall of 10 unrelated words. This word recall task, adapted from the cognitive battery of the Health and Retirement Study (HRS), was originally based on the CERAD Word List Memory Test, which is widely used to assess episodic memory in aging populations [43]. Scores for both immediate and delayed recall ranged from 0 to 10, yielding a composite memory score ranging from 0 to 20, with higher scores indicating better memory performance. The immediate and delayed recall tests have demonstrated good construct validity and consistency [44]. Executive function was assessed using a verbal fluency task, in which individuals were required to orally name as many animals as they could in 60 seconds. This task was administered to all participants. Given its well-documented reliability and validity, this task has been widely used as a robust indicator of executive function in the ELSA population [45]. The total executive function score, based on the number of unique animal names produced, had no fixed upper limit but typically ranged from 0 to 30. Orientation was assessed using date-related questions (one point each for the day of the month, month, year, and day of the week). The total orientation score ranged from 0 to 4. A global cognitive score was calculated as the sum of the individual scores for memory (0–20), executive function (approximately 0–30), and orientation (0–4). In general, higher scores indicate better cognitive function [46].

### 2.4 Covariates

We selected covariates a priori based on their potential to confound the relationship between psychosocial stressors and cognitive outcomes. These included: Demographic variables: Age; sex (female/male); education (below high school, high school, college or above); marital status (never married, married/partnered, separated/divorced/widowed). Lifestyle variables: Smoking status (non-smokers: never smoked or ex-smokers; smokers: current smoking); alcohol consumption (<1 drink/week, ≥1 drink/week); physical activity (sedentary, low, moderate, high); body mass index (BMI: underweight, normal range, overweight, obesity). Health indicators: hypertension (yes, no); diabetes (yes, no); cardiovascular disease (yes, no).

### 2.5 Statistical analyses

First, we described baseline (wave 4) characteristics of the sample using means ± standard deviation for continuous variables and counts with proportions (n, %) for categorical variables. Continuous variables were examined for normality, and transformations (e.g., log-transformation) were applied when distributions were skewed. Second, we examined the baseline associations between psychosocial stressors and cognitive function at wave 4 using multiple linear regression analysis. Psychosocial stressors were categorized into three groups: No Stressors (reference group), One Stressor, and Multiple Stressors (≥2). Cognitive outcomes included global cognitive score, memory score, executive function score, and orientation score, with results reported as regression coefficients (β) and 95 % confidence intervals (CI). Third, to assess the longitudinal associations between psychosocial stressors and cognitive function, we employed a linear mixed-effects model spanning wave 4-9. This analysis evaluated how different trajectories of psychosocial stressors were associated with changes in cognitive function over a 10-year period. β and 95 % CI were reported for each cognitive outcome. For both analyses, Model 1 was unadjusted. Model 2 accounted for age, sex, education, and marital status. Model 3 further adjusted for smoking status, alcohol consumption, physical activity, and BMI category. Model 4 further included hypertension, diabetes, and cardiovascular disease to assess the independent contribution of psychosocial stressors to cognitive outcomes. All analyses were conducted using R 4.2.2.

### 2.6 Sensitivity and subgroup analyses

As the executive function test was not administered in Wave 6, executive scores for this wave were imputed using multiple imputation by chained equations (MICE). Variables used for imputation included baseline demographic characteristics and baseline cognitive scores, and 10 imputations were performed per cohort, generating 10 complete datasets. According to Rubin’s recommendation [47], multiple imputed datasets were generated, and a single longitudinal analysis was conducted with results pooled using R. In addition, missing covariate values were imputed using the MICE procedure. To assess the robustness of our findings and explore potential effect modifications, we conducted a series of sensitivity and subgroup analyses: (1) Robust regression sensitivity analysis: To further evaluate the cross-sectional associations, we applied robust regression techniques, which account for potential outliers and heteroskedasticity in the data. (2) Comparison of random intercept vs. random slope models: To test the robustness of the longitudinal estimates, we compared two modeling approaches: the random intercept model assumes a uniform rate of cognitive decline across individuals, while the random slope model allows for individual-specific differences in cognitive trajectories over time. (3) To examine potential effect modifications, we conducted subgroup analyses by stratifying the sample based on sex, education, marital status, smoking status, alcohol consumption, physical activity, BMI category, hypertension, diabetes and cardiovascular disease.

## 3 Results

### 3.1 Descriptive analysis

To describe the baseline characteristics of the participants and their distribution across different levels of psychosocial stressors, we stratified the 10,893 individuals by the number of reported stressors (0, 1, or ≥2). The corresponding descriptive statistics are summarized in Table 1 and the full frequency distribution of stressor counts can be found in Supplementary Table 1. Overall, 4,720 participants (43.3 %) reported no stressors, 4,193 (38.5 %) reported one stressor, and 1,980 (18.2 %) reported multiple stressors. The mean age of the analytic sample was 64.9 ± 10.1 years, with participants reporting multiple stressors tending to be slightly older (66.5 ± 9.9) compared to those reporting none (63.6 ± 9.9). Women constituted 55.5 % of the total sample, and the proportion of women was notably higher among participants with multiple stressors (62.5 %) than among those with none (52.1 %).

**Table 1:** Baseline characteristics of participants stratified by number of psychosocial stressors.

Characteristics	Total	No Stressors (0)	One Stressor (1)	Multiple Stressors (≥2)
**Reports** (n, %)	10,893	4,720 (43.3 %)	4,193 (38.5 %)	1,980 (18.2 %)
**Age** (mean ± SD)	64.9 ± 10.1	63.6 ± 9.9	65.7 ± 10.3	66.5 ± 9.9
**Sex** (n, %)				
Female	6,042 (55.5 %)	2,458 (52.1 %)	2,347 (56.0 %)	1,237 (62.5 %)
Male	4,851 (44.5 %)	2,262 (47.9 %)	1,846 (44.0 %)	743 (37.5 %)
**Education** (n, %)				
Below high school	3,863 (35.5 %)	1,560 (33.0 %)	1,469 (35.0 %)	834 (42.1 %)
High school	2,078 (19.0 %)	897 (19.0 %)	803 (19.2 %)	378 (19.1 %)
College or above	4,080 (37.5 %)	1,911 (40.5 %)	1,569 (37.4 %)	600 (30.3 %)
Unknown or missing	872 (8.0 %)	352 (7.5 %)	352 (8.4 %)	168 (8.5 %)
**Marital Status** (n, %)				
Never married	554 (5.1 %)	236 (5.0 %)	213 (5.1 %)	105 (5.3 %)
Married or partnered	7,426 (68.2 %)	3,314 (70.2 %)	2,850 (68.0 %)	1,262 (63.7 %)
Separated/divorced/Widowed	2,505 (23.0 %)	974 (20.6 %)	995 (23.7 %)	536 (27.1 %)
Unknown or missing	408 (3.7 %)	196 (4.2 %)	135 (3.2 %)	77 (3.9 %)
**Smoke** (n, %)				
Non-smokers (never smoked or ex-smokers)	9,156 (84.1 %)	3,951 (83.7 %)	3,536 (84.3 %)	1,669 (84.3 %)
Smokers (current smoking)	1,525 (14.0 %)	649 (13.8 %)	590 (14.1 %)	286 (14.4 %)
Unknown or missing	212 (1.9 %)	120 (2.5 %)	67 (1.6 %)	25 (1.3 %)
**Drink** (n, %)				
<1 drink/week	2,957 (27.1 %)	1,148 (24.3 %)	1,165 (27.8 %)	644 (32.5 %)
≥1 drink/week	5,972 (54.8 %)	2,700 (57.2 %)	2,274 (54.2 %)	998 (50.4 %)
Unknown or missing	1,964 (18.0 %)	872 (18.5 %)	754 (18.0 %)	338 (17.1 %)
**Physical activity** (n, %)				
Sedentary	751 (6.9 %)	293 (6.2 %)	300 (7.2 %)	158 (8.0 %)
Low	2,524 (23.2 %)	963 (20.4 %)	1,047 (25.0 %)	514 (26.0 %)
Moderate	5,357 (49.2 %)	2,320 (49.2 %)	2,065 (49.2 %)	972 (49.1 %)
High	2,252 (20.7 %)	1,140 (24.2 %)	776 (18.5 %)	336 (17.0 %)
Unknown or missing	9 (0.1 %)	4 (0.1 %)	5 (0.1 %)	0 (0.0 %)
**BMI** (n, %)				
Underweight	69 (0.6 %)	29 (0.6 %)	28 (0.7 %)	12 (0.6 %)
Normal range	2,146 (19.7 %)	1,001 (21.2 %)	818 (19.5 %)	327 (16.5 %)
Overweight	3,425 (31.4 %)	1,476 (31.3 %)	1,351 (32.2 %)	598 (30.2 %)
Obesity	2,577 (23.7 %)	976 (20.7 %)	1,020 (24.3 %)	581 (29.3 %)
Unknown or missing	2,676 (24.6 %)	1,238 (26.2 %)	976 (23.3 %)	462 (23.3 %)
**Hypertension** (n, %)				
Yes	5,086 (46.7 %)	1,970 (41.7 %)	2,051 (48.9 %)	1,065 (53.8 %)
No	5,801 (53.3 %)	2,748 (58.2 %)	2,139 (51.0 %)	914 (46.2 %)
Unknown or missing	6 (0.1 %)	2 (0.1 %)	3 (0.1 %)	1 (0.1 %)
**Diabetes** (n, %)				
Yes	1,137 (10.4 %)	313 (6.6 %)	505 (12.1 %)	319 (16.1 %)
No	9,749 (89.5 %)	4,405 (93.3 %)	3,683 (87.8 %)	1,661 (83.9 %)
Unknown or missing	7 (0.1 %)	2 (0.1 %)	5 (0.1 %)	0 (0.0 %)
**Heart disease** (n, %)				
Yes	1747 (16.0 %)	615 (13.0 %)	731 (17.4 %)	401 (20.3 %)
No	9144 (83.9 %)	4,105 (87.0 %)	3,460 (82.5 %)	1,579 (79.7 %)
Unknown or missing	2 (0.1 %)	0 (0.0 %)	2 (0.1 %)	0 (0.0 %)
**Scores** (mean ± SD)				
Global cognitive score	35.0 ± 9.4	35.6 ± 9.3	34.7 ± 9.5	34.1 ± 9.0
Memory score	10.4 ± 3.8	10.6 ± 3.9	10.3 ± 3.9	10.2 ± 3.7
Executive function score	20.8 ± 7.0	21.3 ± 7.0	20.6 ± 7.0	20.2 ± 6.7
Orientation score	3.8 ± 0.5	3.8 ± 0.5	3.8 ± 0.6	3.8 ± 0.5

Values are presented as n ( %) for categorical variables and mean ± standard deviation (SD) for continuous variables.

Educational attainment varied across groups: 30.3 % of those with multiple stressors had college or above education, compared to 40.5 % in the no-stressor group. In terms of marital status, 63.7 % of those with multiple stressors were married or partnered, whereas 70.2 % of those without any stressors were married or partnered. Lifestyle factors also differed by stressor burden. Although most participants were non-smokers (84.1 %), a slightly higher percentage of current smokers was observed among those with multiple stressors (14.4 %) than among those with no stressors (13.8 %). The majority of participants (54.8 %) reported drinking alcohol at least once per week, yet participants with multiple stressors were more likely to consume alcohol less frequently (<1 drink/week). Regarding physical activity, 8.0 % of those with multiple stressors were classified as sedentary, compared to 6.2 % in the no-stressor group.

Cardiometabolic conditions were generally more prevalent in the multiple-stressor group. For instance, hypertension was reported by 53.8 % of those with multiple stressors (vs. 41.7 % in the no-stressor group), and diabetes was notably higher in the multiple-stressor group (16.1 %) compared to those without stressors (6.6 %). Similarly, cardiovascular disease was more common among those with multiple stressors (20.3 % vs. 13.0 % in the no-stressor group).

Global cognitive scores (mean ± SD) were 35.6 ± 9.3, 34.7 ± 9.5, and 34.1 ± 9.0 for participants reporting no stressors, one stressor, and multiple stressors, respectively. In line with this, both memory and executive function scores showed a slightly lower mean among the multiple-stressor group compared to those with no stressors. In contrast, orientation scores remained relatively similar across groups (3.8 ± 0.5 overall).

### 3.2 Baseline associations between psychosocial stressors and cognitive function

To examine the associations between psychosocial stressors and cognitive performance, we conducted multiple linear regression analyses across three cognitive domains—memory, executive function, and orientation—as well as a global cognition score. Baseline associations across these cognitive measures were summarized in Table 2. In the unadjusted model (Model 1), psychosocial stressors were significantly associated with lower scores across all cognitive domains. Compared to individuals with no stressors, those with one or more stressors had significantly lower global cognition (one stressor: β = -0.955, 95 % CI [-1.343, -0.567], p < 0.001; two or more: β = -1.541, 95 % CI [-2.031, -1.051], p < 0.001), memory (β = -0.293, 95 % CI [-0.454, -0.132], p < 0.001; β = -0.392, 95 % CI [-0.594, -0.190], p < 0.001), executive function (β = -0.630, 95 % CI [-0.920, -0.340], p < 0.001; β = -1.123, 95 % CI [-1.490, -0.756], p < 0.001), and orientation scores (two or more stressors: β = -0.093, 95 % CI [-0.175, -0.011], p < 0.01). After adjusting for demographic factors (Model 2) and further controlling for lifestyle factors (Model 3), the associations between psychosocial stressors and memory scores as well as orientation scores became non-significant, suggesting that these relationships were largely accounted for by demographic and health-related variables (covariate–outcome associations were detailed in Supplementary Table 2). However, global cognitive scores and executive function scores remained significantly associated with two or more psychosocial stressors across all three models. Supplementary Table 3 showed that the robust regression analysis yielded stable results, further validating the observed associations.

**Table 2:** Baseline association between psychosocial stressors and cognitive function: multiple linear regression analysis.

**Model**	Stressors	Global cognitive score	Memory score	Executive function score	Orientation score
**Model 1**	1 vs 0	-0.96 (-1.34 – -0.57) p < 0.001^⁎⁎⁎^	-0.29 (-0.45 – -0.13) p < 0.001^⁎⁎⁎^	-0.63 (-0.92 – -0.34) p < 0.001^⁎⁎⁎^	-0.03 (-0.10 – 0.03) p = 0.317
	≥2 vs 0	-1.54 (-2.03 – -1.05) p < 0.001**^⁎⁎⁎^**	-0.39 (-0.59 – -0.19) p < 0.001^⁎⁎⁎^	-1.12 (-1.49 – -0.76) p < 0.001^⁎⁎⁎^	-0.09 (-0.18 – -0.01) p < 0.01^⁎⁎^
**Model 2**	1 vs 0	-0.25 (-0.61 – 0.12) p = 0.180	-0.01 (-0.16 – 0.14) p = 0.864	-0.22 (-0.50 – 0.06) p = 0.118	0.02 (-0.04 – 0.09) p = 0.525
	≥2 vs 0	-0.53 (-0.99 – -0.08) p < 0.01**^⁎⁎^**	0.01 (-0.18 – 0.20) p = 0.934	-0.54 (-0.89 – -0.19) p < 0.001^⁎⁎⁎^	-0.02 (-0.10 – 0.07) p = 0.686
**Model 3**	1 vs 0	-0.26 (-0.62 – 0.11) p = 0.166	-0.04 (-0.19 – 0.11) p = 0.590	-0.20 (-0.48 – 0.08) p = 0.155	0.02 (-0.05 – 0.08) p = 0.565
	≥2 vs 0	-0.55 (-1.01 – -0.09) p < 0.01**^⁎⁎^**	-0.06 (-0.25 – 0.13) p = 0.518	-0.49 (-0.84 – -0.14) p < 0.001^⁎⁎⁎^	-0.02 (-0.10 – 0.06) p = 0.617
**Model 4**	1 vs 0	0.06 (-0.36 – 0.48) p = 0.785	-0.03 (-0.20 – 0.13) p = 0.700	0.11 (-0.22 – 0.45) p = 0.509	0.04 (-0.03 – 0.12) p = 0.270
	≥2 vs 0	-0.08 (-0.62 – 0.45) p = 0.761	-0.08 (-0.29 – 0.13) p = 0.483	0.02 (-0.41 – 0.45) p = 0.927	-0.03 (-0.13 – 0.07) p = 0.589

A total of 10,893 participants were included in this cross-sectional analysis. Results are presented as regression coefficients (β) and 95 % confidence intervals (CI). “No stressor” group was used as the reference category. The exposure variable represents the number of psychosocial stressors experienced (0, 1, or ≥2). Comparisons such as “1 vs 0” and “≥2 vs 0” indicate the baseline differences in cognitive function between participants exposed to one or multiple stressors, respectively, and those with no stressors.

Model 1 was unadjusted.

Model 2 additionally adjusts for age and sex, education, marital status.

Model 3 additionally adjusts for smoking, alcohol consumption, physical activity and BMI category.

Model 4 additionally adjusts for hypertension, diabetes, and cardiovascular disease.

Missing covariate values were handled using multiple imputation with chained equations.

This table presents baseline associations between psychosocial stressor levels and cognitive function at Wave 4. It provides a detailed overview of how differing levels of psychosocial stress are related to cognitive performance in a cross-sectional context.

*p < 0.05; **p < 0.01; *** *p* < 0.001

### 3.3 Longitudinal associations between psychosocial stressors and cognitive function

To evaluate the longitudinal impact of psychosocial stressors on cognitive function over a 10-year follow-up period (waves 4–9), we applied linear mixed-effects models. The estimated associations are summarized in Table 3. The results show that individuals exposed to higher levels of psychosocial stressors experienced more rapid cognitive decline over time.

**Table 3:** Longitudinal association between psychosocial stressors and cognitive function across waves: linear mixed-effects model analysis.

Model	Stressors	Global cognitive score	Memory score	Executive function score	Orientation score
**Model 1**	1 vs 0	-0.16 (-0.23 – -0.09) p < 0.001^⁎⁎⁎^	-0.08 (-0.11 – -0.05) p < 0.001^⁎⁎⁎^	-0.07 (-0.12 – -0.01) p = 0.015	-0.02 (-0.03 – -0.00) p = 0.005
	≥2 vs 0	-0.35 (-0.44 – -0.26) p < 0.001**^⁎⁎⁎^**	-0.15 (-0.19 – -0.11) p < 0.001^⁎⁎⁎^	-0.18 (-0.25 – -0.11) p < 0.001^⁎⁎⁎^	-0.02 (-0.03 – -0.00) p = 0.010
**Model 2**	1 vs 0	-0.17 (-0.25 – -0.10) p <0.001**^⁎⁎⁎^**	-0.08 (-0.11 – -0.05) p < 0.001^⁎⁎⁎^	-0.07 (-0.13 – -0.02) p = 0.013	-0.02 (-0.03 – -0.01) p = 0.003
	≥2 vs 0	-0.35 (-0.45 – -0.26) p <0.001**^⁎⁎⁎^**	-0.15 (-0.19 – -0.10) p < 0.001^⁎⁎⁎^	-0.18 (-0.25 – -0.11) p < 0.001^⁎⁎⁎^	-0.02 (-0.04 – -0.01) p = 0.003
**Model 3**	1 vs 0	-0.17 (-0.26 – -0.09) p <0.001**^⁎⁎⁎^**	-0.06 (-0.10 – -0.02) p = 0.003	-0.10 (-0.17 – -0.03) p = 0.004	-0.01 (-0.03 – -0.00) p = 0.045
	≥2 vs 0	-0.34 (-0.45 – -0.23) p <0.001**^⁎⁎⁎^**	-0.12 (-0.16 – -0.07) p < 0.001^⁎⁎⁎^	-0.20 (-0.29 – -0.11) p < 0.001^⁎⁎⁎^	-0.03 (-0.05 – -0.01) p = 0.002
**Model 4**	1 vs 0	-0.17 (-0.26 – -0.09) p <0.001**^⁎⁎⁎^**	-0.06 (-0.10 – -0.02) p = 0.003	-0.10 (-0.17 – -0.03) p = 0.004	-0.01 (-0.03 – -0.00) p = 0.045
	≥2 vs 0	-0.34 (-0.45 – -0.23) p <0.001**^⁎⁎⁎^**	-0.12 (-0.16 – -0.07) p < 0.001^⁎⁎⁎^	-0.20 (-0.29 – -0.11) p < 0.001^⁎⁎⁎^	-0.03 (-0.05 – -0.01) p = 0.002

A total of 10,893 participants were included in the analysis. Results are presented as regression coefficients (β) and 95 % confidence intervals (CI). "No stressor" group was used as the reference category. The exposure variable represents the number of psychosocial stressors (0, 1, or ≥2), and the interaction terms (e.g., "1 vs 0", "≥2 vs 0") reflect the moderating effect of stressor levels on cognitive trajectories over time, which refers to years since baseline (Year 0 = Wave 4, 2008–2009).

Model 1 was unadjusted.

Model 2 additionally adjusts for age and sex, education, marital status.

Model 3 additionally adjusts for smoking, alcohol consumption, physical activity and BMI category.

Model 4 additionally adjusts for hypertension, diabetes, and cardiovascular disease.

Missing covariate values were handled using multiple imputation with chained equations.

This table presents longitudinal associations between psychosocial stressors and cognitive function over a 10-year follow-up period.

***p < 0.001

In the unadjusted model (Model 1), individuals experiencing one or more psychosocial stressors exhibited significantly greater cognitive decline compared to those with no stressors. Specifically, individuals with multiple stressors had steeper declines in global cognition (β = -0.35, 95 % CI [-0.44, -0.26], p < 0.001), memory (β = -0.15, 95 % CI [-0.19, -0.11], p < 0.001), and executive function (β = -0.18, 95 % CI [-0.25, -0.11], p < 0.001). The presence of a single stressor was also associated with significant declines in global cognition (β = -0.16, 95 % CI [-0.23, -0.09], p < 0.001), memory (β = -0.08, 95 % CI [-0.11, -0.05], p < 0.001), although with smaller effect sizes.

After adjusting for demographic factors (Model 2) and lifestyle variables (Model 3), the associations remained largely consistent, with multiple stressors continuing to be significantly associated with declines in all cognitive domains. In the fully adjusted model (Model 4), which accounted for chronic health conditions, the negative effects of multiple stressors on global cognition (β = -0.34, 95 % CI [-0.45, -0.23], p < 0.001), memory (β = -0.12, 95 % CI [-0.16, -0.07], p < 0.001), and executive function (β = -0.20, 95 % CI [-0.29, -0.11], p < 0.001) remained statistically significant. These findings highlight the robust and cumulative impact of psychosocial stress on cognitive aging, particularly among those exposed to multiple stressors, with effects remaining significant after adjusting for key demographic, lifestyle, and health-related factors.

### 3.4 Comparison of random intercept and random slope models

To further assess the robustness of the association between psychosocial stressors and cognitive decline, we fitted both random intercept and random slope models. The corresponding results are presented in Table 4. In both models, the presence of psychosocial stressors was significantly associated with a steeper cognitive decline over time. Compared to individuals with no stressors, those experiencing a single stressor showed a significant decline in cognitive function as the waves progressed (random intercept model: β = -0.17, 95 % CI [-0.26, -0.09], p < 0.001; random slope model: β = -0.17, 95 % CI [-0.27, -0.08], p < 0.001). The effect became more pronounced with multiple stressors, leading to a greater cognitive decline over successive waves (random intercept model: β = -0.34, 95 % CI [-0.45, -0.23], p < 0.001; random slope model: β = -0.34, 95 % CI [-0.46, -0.22], p < 0.001). These findings suggest a dose-dependent effect, where greater exposure to stressors accelerates cognitive deterioration over time. The similar results observed across both models further support the robustness of the association.

**Table 4:** Longitudinal association between psychosocial stressors and cognitive function: a comparison of random intercept and random slope models across demographic subgroups.

	*Random Intercept Model*			*Random Slope Model*		
*Predictors*	β	95 % CI	p	β	95 % CI	p
**Stressors**						
1 vs 0	-0.17	-0.26 – -0.09	<0.001**^⁎⁎⁎^**	-0.17	-0.27 – -0.08	<0.001**^⁎⁎⁎^**
≥2 vs 0	-0.34	-0.45 – -0.23	<0.001**^⁎⁎⁎^**	-0.34	-0.46 – -0.22	<0.001**^⁎⁎⁎^**
**Age**	-0.37	-0.39 – -0.35	<0.001**^⁎⁎⁎^**	-0.32	-0.34 – -0.30	<0.001**^⁎⁎⁎^**
**Sex** (Ref: Female)						
Male	-1.46	-1.83 – -1.09	<0.001**^⁎⁎⁎^**	-1.48	-1.84 – -1.12	<0.001**^⁎⁎⁎^**
**Education** (Ref: College or above)						
High school	-1.47	-1.94 – -1.00	<0.001**^⁎⁎⁎^**	-1.66	-2.11 – -1.21	<0.001**^⁎⁎⁎^**
Below high school	-5.11	-5.54 – -4.67	<0.001**^⁎⁎⁎^**	-5.21	-5.63 – -4.79	<0.001**^⁎⁎⁎^**
**Marital status** (Ref: Married or partnered)						
Never married	-0.11	-0.57 – 0.35	0.637	-0.07	-0.51 – 0.38	0.763
Separated/divorced/Widowed	-1.46	-2.28 – -0.64	<0.001**^⁎⁎⁎^**	-1.39	-2.18 – -0.60	0.001
**Smoke** (Ref: non-smokers [never smoked or ex-smokers])						
smokers (current smoking)	-0.90	-1.46 – -0.34	0.002	-0.79	-1.33 – -0.26	0.004
**Alcohol consumption** (Ref: <1 drink/week)						
≥1 drink/week	1.40	1.00 – 1.80	0.002	1.39	1.00 – 1.77	0.003
**Physical activity** (Ref: High)						
Moderate	-1.02	-1.47 – -0.56	<0.001**^⁎⁎⁎^**	-0.93	-1.37 – -0.50	<0.001**^⁎⁎⁎^**
Low	-2.59	-3.15 – -2.02	<0.001**^⁎⁎⁎^**	-2.45	-3.00 – -1.91	<0.001**^⁎⁎⁎^**
Sedentary	-4.14	-5.14 – -3.14	<0.001**^⁎⁎⁎^**	-3.97	-4.93 – -3.00	<0.001**^⁎⁎⁎^**
**BMI category** (Ref: Normal range)						
Underweight	-1.49	-3.52 – 0.54	0.150	-1.68	-3.64 – 0.29	0.094
Overweight	0.25	-0.24 – 0.74	0.311	0.24	-0.19 – 0.67	0.279
Obesity	0.28	-0.17 – 0.72	0.224	0.23	-0.24 – 0.70	0.340
**Hypertension** (Ref: No)						
Yes	-0.53	-0.90 – -0.15	0.006	-0.45	-0.81 – -0.09	0.014
**Diabetes** (Ref: No)						
Yes	-0.75	-1.34 – -0.16	0.013	-0.83	-1.41 – -0.26	0.004
**Cardiovascular disease** (Ref: No)						
Yes	-0.16	-0.65 – 0.34	0.543	-0.12	-0.60 – 0.36	0.628

A total of 10,893 participants were included in the analysis. Results of the regression analysis are reported as regression coefficients (β) and 95 % confidence intervals (CI). Ref: reference.

The exposure variable represents the number of psychosocial stressors (0, 1, or ≥2), with the “no stressor” group as the reference category. The interaction terms (e.g., “1 vs 0”, “≥2 vs 0”) indicate how different levels of stressor exposure are associated with changes in cognitive trajectories over time, which refers to years since baseline (Year 0 = Wave 4, 2008–2009). Missing covariate values were handled using multiple imputation with chained equations

Both random intercept and random slope models were used to evaluate the longitudinal effects of psychosocial stressors on cognitive function. The random intercept model assumes a uniform slope across all individuals, allowing only individual-specific variation at the baseline level (intercept). In contrast, the random slope model enables each individual to have a distinct slope across follow-up waves, offering a more comprehensive depiction of inter-individual differences over time. By comparing these two models in a sensitivity analysis and incorporating between-group comparisons, this approach provides a more accurate assessment of model performance and the heterogeneity in cognitive trajectories among different populations.

***p < 0.001

### 3.5 Subgroup analysis

Subgroup analysis showed that several demographic and lifestyle factors were significantly associated with cognitive function (Table 4). Older age (β = -0.37, 95 % CI [-0.39, -0.35], p < 0.001) was linked to lower cognitive scores and male participants exhibited lower cognitive scores than females (β = -1.46, 95 % CI [-1.83, -1.09], p < 0.001). Lower educational attainment was strongly associated with worse cognitive performance. Individuals with high school education had significantly lower cognitive scores than those with a college degree or above (β = -1.47, 95 % CI [-1.94, -1.00], p < 0.001), while those with below high school education showed the greatest cognitive disadvantage (β = -5.11, 95 % CI [-5.54, -4.67], p < 0.001). Marital status also influenced cognitive function. Compared to those who were married or partnered, individuals who were separated, divorced, or widowed had significantly lower cognitive scores (β = -1.46, 95 % CI [-2.28, -0.64], p < 0.001). Among lifestyle factors, lower physical activity levels (β = -2.59, 95 % CI [-3.15, -2.02], p < 0.001), and sedentary behavior (β = -4.14, 95 % CI [-5.14, -3.14], p < 0.001) were significantly associated with poorer cognitive function.

## 4 Discussion

In this prospective cohort study of older English adults, we investigated how different burdens of psychosocial stressors—encompassing financial strain, caregiving, disability, and limiting long-term illness—relate to both baseline cognitive performance and longitudinal change over a 10-year period. Our findings indicate that higher cumulative stressor burdens are associated with lower baseline cognitive scores and a steeper rate of decline, particularly in global cognition and executive function. These associations persisted even after extensive adjustment for demographic, lifestyle, and clinical factors, highlighting the importance of psychosocial stress as a potentially modifiable risk factor for adverse cognitive trajectories.

Several key observations emerged from our analyses. First, individuals reporting multiple psychosocial stressors demonstrated consistently worse cognitive performance at baseline. Although the association between stressors and memory performance was attenuated after adjusting for demographic and health-related covariates, it remained robust in longitudinal models, suggesting that the negative impact of stress may become more apparent over time. Moreover, a dose-dependent trend emerged: those with one stressor experienced modest, though significant, declines in cognitive function, whereas the presence of two or more stressors yielded more pronounced deterioration. This gradient underscore the cumulative burden that psychosocial adversity can impose on cognitive health. Second, our comparison of random intercept and random slope models yielded highly consistent estimates, reinforcing the robustness of our findings. Both modeling strategies confirmed that the burden of psychosocial stress predicts faster cognitive decline, thus reducing the likelihood that our results were driven by modeling assumptions. Lastly, subgroup analyses suggested that the detrimental effects of multiple stressors were broadly observed across demographic strata. Although sex and education were strongly associated with overall cognitive level—elderly males under stress [[48], [49], [50]] and those with lower educational attainment [51] performing worse on average—greater stress exposure continued to predict steeper cognitive declines even after accounting for these and other sociodemographic factors.

In line with our findings, investigations of older adults in diverse cohorts have similarly reported that financial strain, caregiving responsibilities, and other stressors correlate with poorer cognitive function and memory decline over time [[52], [53], [54], [55]]. For instance, a recent meta-analysis involving over 110,000 individuals confirmed that financial scarcity exerts a moderate but consistent detrimental effect on cognitive performance, particularly among those with lower educational attainment and prolonged exposure to financial hardship [52]. In parallel, caregiving-related stress has also been found to negatively affect psychological well-being and mental resilience. Cross-sectional studies from China and the United States revealed that stress burden among caregivers was significantly associated with increased psychological distress, highlighting the complex pathways through which chronic stress may impair emotional and cognitive health [53,56]. Several mechanisms might underlie these associations. Chronic or repeated stress can trigger neuroendocrine imbalances, leading to persistently elevated cortisol levels that may exert neurotoxic effects on brain regions critical for memory and executive function, such as the hippocampus and prefrontal cortex [57]. Additionally, psychosocial adversity is known to exacerbate inflammatory processes, contributing to neuroinflammation that can accelerate neuropathological changes [58]. Heightened cardiovascular reactivity due to stress, coupled with elevated rates of conditions such as hypertension and diabetes in stressed individuals, may also compromise cerebrovascular integrity, further impacting cognitive function [7,59,60]. In our cohort, participants with multiple stressors had a higher prevalence of chronic conditions (e.g., hypertension, diabetes), potentially compounding the detrimental effects on brain health.

A major strength of this study lies in its use of a large, nationally representative cohort followed for up to a decade. The repeated measures of multiple cognitive domains—including memory, executive function, and orientation—enabled a nuanced assessment of how stressor profiles might differentially affect specific cognitive processes. Moreover, we adopted robust analytic strategies, including a comparison of random intercept versus random slope models, multiple imputations to handle missing data, and extensive covariate adjustments. These approaches help mitigate potential biases stemming from attrition, measurement error, and unobserved confounding.

Nevertheless, some limitations merit consideration. First, the psychosocial stressor measures were based on self-report, which may introduce reporting bias. Additionally, while our binary categorization of stressors (presence vs. absence) provided a practical approach to classifying risk, it may oversimplify the complexity of how older adults experience and perceive stress. Future studies incorporating more granular assessments of stress duration and severity could yield deeper insights. We also cannot fully exclude the possibility of reverse causation, as cognitive deficits may influence the reporting of stress or the ability to cope with it. However, our longitudinal design and exclusion of individuals with diagnosed dementia at baseline help mitigate this concern. Furthermore, while the cognitive tasks used in this study are widely recognized and validated in aging research, some may be subject to practice effects and limited sensitivity to subtle changes over time. Test-retest reliability may also vary across cognitive domains, which could influence the interpretation of longitudinal trends. Lastly, despite the nationally representative nature of the ELSA, our findings may not be fully generalizable to other cultural or socioeconomic contexts where stressors manifest differently.

## 5 Conclusion

These findings underscore the importance of recognizing and mitigating psychosocial stressors to preserve cognitive function in later life. Our results suggest that cumulative psychosocial adversity can significantly erode both baseline cognitive performance and the capacity to maintain cognitive health over time. Given the modifiable nature of many stressors—such as financial strain and caregiving burdens—interventions aiming to alleviate stress in vulnerable subgroups may hold promise for preventing or delaying cognitive decline. Future work should explore whether targeted stress-management programs, social support interventions, or policy measures that address financial insecurity can effectively reduce cognitive risks. As populations continue to age globally, elucidating strategies to mitigate the toll of chronic stress on cognitive outcomes will be an essential public health priority.

## Funding

This work was supported by funding from Alzheimer Nederland (WE.03-2024-20, Ulrich L. M. Eisel and Jocelien D. A. Olivier) and the National Natural Science Foundation of China (NSFC 82401393, Feng Zhang).

## Ethical approval

All participants provided written informed consent, and ethical approval was obtained from the National Research Ethics Service (London Multicentre Research Ethics Committee).

## Data availability

The datasets analyzed during the current study are available from the English Longitudinal Study of Ageing repository (https://www.elsa-project.ac.uk/) upon reasonable request and application to the data custodians. All data generated or analyzed in this study are included in this published article.

## Declaration of generative AI and AI-assisted technologies in the writing process

No generative AI and AI-assisted technologies were used in the writing process of this work.

## CRediT authorship contribution statement

**Jiahao Li:** Writing – review & editing, Writing – original draft, Methodology, Formal analysis, Data curation, Conceptualization. **Natalia Ortí-Casañ:** Writing – review & editing. **Irem Bayraktaroglu:** Writing – review & editing. **Giulia Mozzanica:** Writing – review & editing. **Feng Zhang:** Writing – review & editing, Supervision, Conceptualization. **Jocelien D.A. Olivier:** Writing – review & editing, Supervision. **Ulrich L.M. Eisel:** Writing – review & editing, Supervision, Project administration, Conceptualization.

## Declaration of competing interest

The authors declare that they have no known competing financial interests or personal relationships that could have appeared to influence the work reported in this paper.

## Appendix Supplementary materials

Image, application 1
