# Supplementary material for: Psychosocial stressors and cognitive function: An analysis using data from the English longitudinal study of ageing
Source: J Prev Alzheimers Dis. 2025 Jun 17;12(8):100232. doi: 10.1016/j.tjpad.2025.100232 (PMC12413730; doi:10.1016/j.tjpad.2025.100232)
Supplement: Supplementary file 1 [file mmc1.doc]

**Supplementary Table 1.** Distribution of total psychosocial stressors

| **Number of Psychosocial Stressors** | **N** | **%** |
| --- | --- | --- |
| 0 (No stressors) | 4,720 | 43.3% |
| 1 | 4,193 | 38.5% |
| 2 | 1,514 | 13.9% |
| 3 | 450 | 4.1% |
| 4 | 16 | 0.1% |
| Total | 10,893 | 100% |

**Supplementary Table 2.** Association between adjusted covariates and global cognitive score at baseline

| *Predictors* | β (95% CI) | p |
| --- | --- | --- |
| **Age** | -0.26 (-0.28 – -0.23) | <0.001**^***^** |
| **Sex** (Ref: Female) |  |  |
| Male | -1.31 (-1.71 – -0.92) | <0.001**^***^** |
| **Education** (Ref: College or above) |  |  |
| High school | -1.98 (-2.48 – -1.48) | <0.001**^***^** |
| Below high school | -5.35 (-5.81 – -4.89) | <0.001**^***^** |
| **Marital status** (Ref: Married or partnered) |  |  |
| Never married | -0.01 (-0.50 – 0.48) | 0.960 |
| Separated/divorced/Widowed | -1.73 (-2.60 – -0.85) | <0.001**^***^** |
| **Smoke** (Ref: non-smokers [never smoked or ex-smokers]) |  |  |
| smokers (current smoking) | -0.48 (-1.08 – 0.11) | 0.111 |
| **Alcohol consumption** (Ref: <1 drink/week) |  |  |
| ≥1 drink/week | 1.34 (0.91 – 1.77) | 0.001 |
| **Physical activity** (Ref: High) |  |  |
| Moderate | -0.75 (-1.23 – -0.27) | 0.002 |
| Low | -2.04 (-2.64 – -1.43) | <0.001**^***^** |
| Sedentary | -3.00 (-4.06 – -1.93) | <0.001**^***^** |
| **BMI category** (Ref: Normal range) |  |  |
| Underweight | -1.61 (-3.78 – 0.56) | 0.147 |
| Overweight | 0.03 (-0.44 – 0.50) | 0.908 |
| Obesity | 0.13 (-0.40 – 0.65) | 0.639 |
| **Hypertension** (Ref: No) |  |  |
| Yes | -0.28 (-0.68 – 0.12) | 0.165 |
| **Diabetes** (Ref: No) |  |  |
| Yes | -0.81 (-1.44 – -0.17) | 0.015 |
| **Cardiovascular disease** (Ref: No) |  |  |
| Yes | -0.09 (-0.63 – 0.44) | 0.730 |

*** *p* < 0.001

**Supplementary Table 3.** Cross-sectional sensitivity analysis of psychosocial stressors on cognitive function

|  | **Stressors** | **Basic Model** | **Robust Regression Sensitivity** |
| --- | --- | --- | --- |
| **Model 1** | 1 vs 0 | -0.96 (-1.34 – -0.57)  p < 0.001**^***^** | -0.93 (-1.31 – -0.55)  p < 0.001**^***^** |
|  | ≥2 vs 0 | -1.54 (-2.03 – -1.05)  p < 0.001**^***^** | -1.60 (-2.09 – -1.12)  p < 0.001**^***^** |
| **Model 2** | 1 vs 0 | 0.06 (-0.36 – 0.48)  p = 0.785 | 0.09 (-0.32 – 0.50)  p = 0.668 |
|  | ≥2 vs 0 | -0.08 (-0.62 – 0.45)  p = 0.761 | -0.02 (-0.54 – 0.50)  p = 0.940 |

A total of 10,893 participants were included in this cross-sectional analysis. Results are presented as regression coefficients (β) and 95% confidence intervals (CI). “No stressor” group was used as the reference category. The exposure variable represents the number of psychosocial stressors experienced (0, 1, or ≥2). Comparisons such as “1 vs 0” and “≥2 vs 0” indicate the baseline differences in cognitive function between participants exposed to one or multiple stressors, respectively, and those with no stressors.

Model 1 was unadjusted.

Model 2 additionally adjusts for age and sex, education, marital status, smoking, alcohol consumption, physical activity, BMI category, hypertension, diabetes, and cardiovascular disease.

*** *p* < 0.001
